# Howship-Romberg Sign and Bowel Obstruction: A Case Report

**DOI:** 10.7759/cureus.5066

**Published:** 2019-07-02

**Authors:** Rasha Saeed, Mohamed Ahmed, Gustavo Lara, Ahmed Mahmoud, Harvey Nurick

**Affiliations:** 1 Surgery, Arrowhead Regional Medical Center, Fontana, USA; 2 Surgery, University of California, Riverside, USA; 3 Surgery, Riverside Community Hospital, Riverside, USA

**Keywords:** obturator hernia, small bowel obstruction, howship-romberg sign

## Abstract

Small bowel obstruction (SBO) is associated with high morbidity and mortality in acute care surgery practice, with hernias constituting a significant portion of the underlying etiology. Physical examination and maneuvers such as changing the extremity position of the patient can further improve clinician's diagnostic acumen to identify the cause of the disease. We present a case of SBO in an 83-year-old female whose physical exam was consistent with an underlying obturator hernia.

## Introduction

The Howship-Romberg sign is an indication of obturator nerve irritation resulting in inner thigh pain that may extend to the knee on internal rotation of the hip [1]. Obturator hernias are rare but pose a diagnostic dilemma with relatively high morbidity and mortality. It is often seen in elderly, thin females who present with signs of small bowel obstruction (SBO) and inner thigh pain [2]. Our patient is an elderly, thin female with an initial evaluation concerning for degenerative joint disease, and further evaluation revealed a strangulated small bowel secondary to an obturator hernia.

## Case presentation

**Figure 1:**
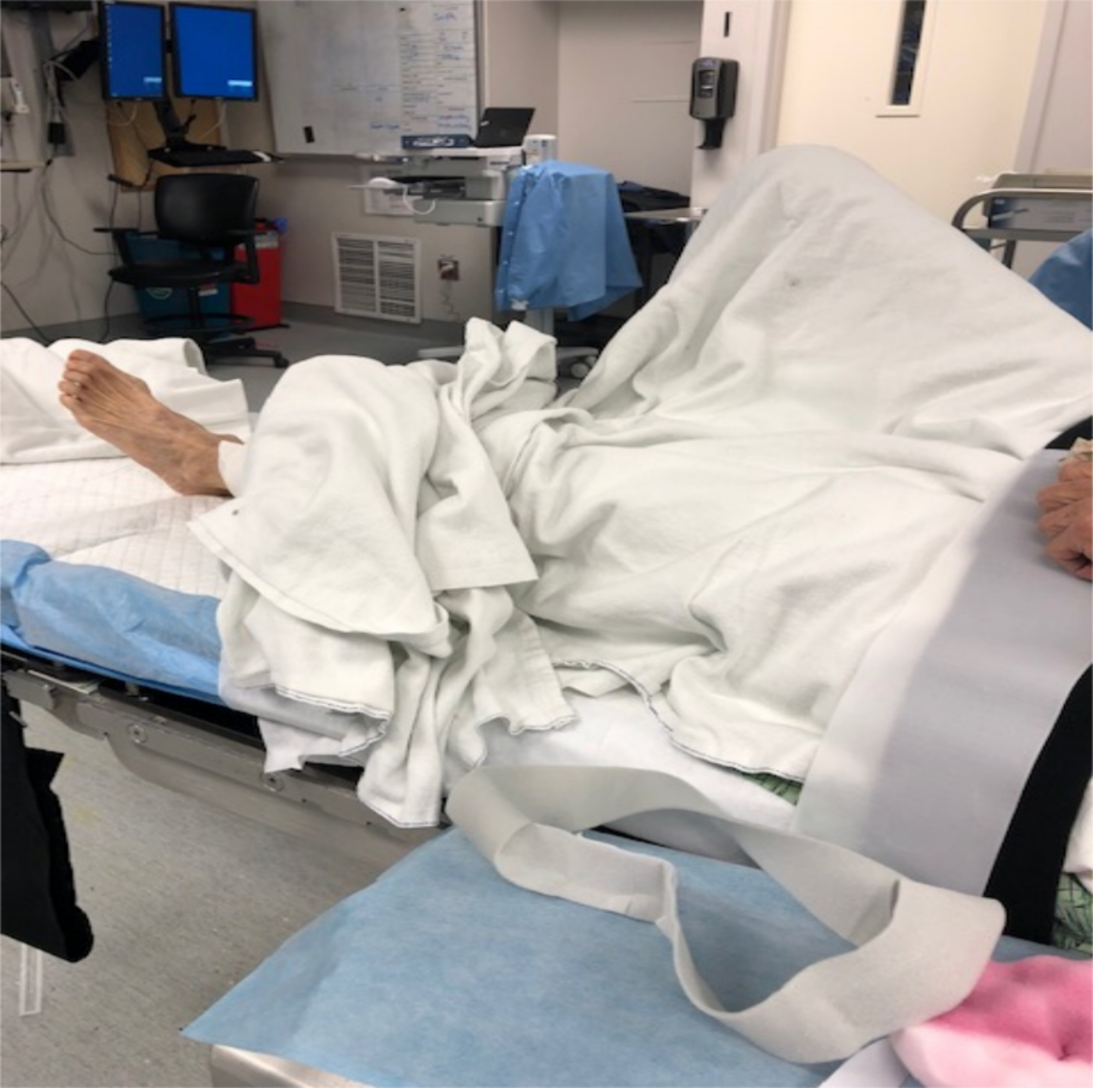
Patient in a comfortable position The patient kept her right hip flexed to minimize her pain.

An 83-year-old female patient presented to our emergency room with a two-day history of right thigh and knee pain, nausea, and obstipation. The initial evaluation was concerning for hip pathology, as she was avoiding right hip extension (Figure 1). Her family reported a significant weight loss over the last year attributed to grieving over her son's death.

**Figure 2:**
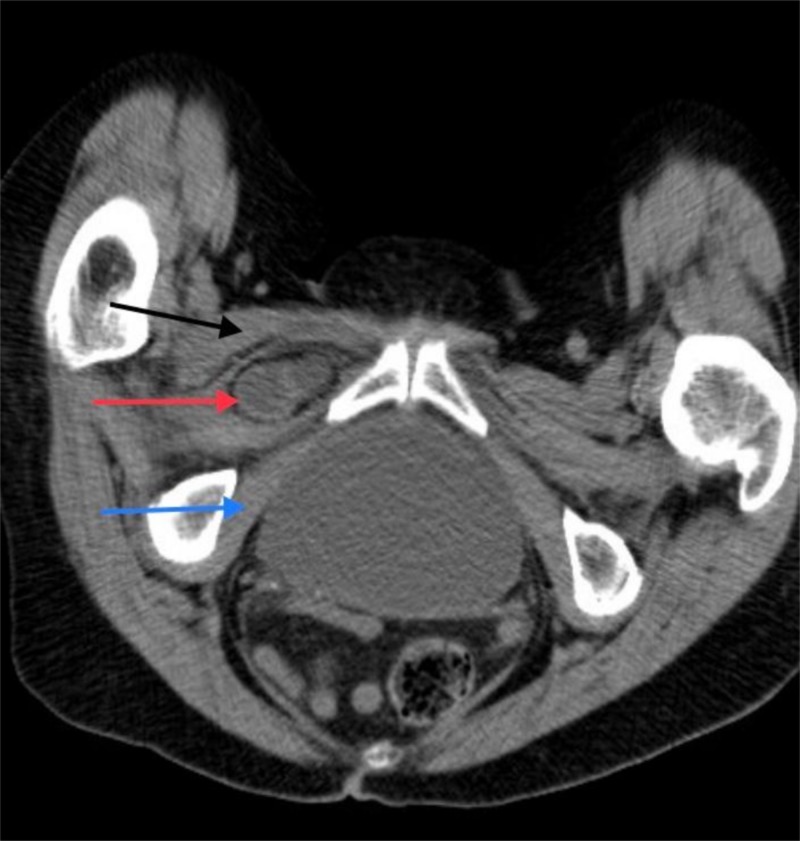
CT scan pelvis Pectineus muscle (black arrow); small bowel (red arrow); obturator muscle (blue arrow)

Her laboratory findings were within normal limits, except for elevated blood urea nitrogen 40 mg/dl (normal range: 7-18) and creatinine 1.48 mg/dl (normal range: 0.55-1.02), consistent with dehydration. A plain X-ray of her abdomen and pelvis revealed mild hip degenerative disease but raised concerns for dilated small bowel loops. A computed tomography (CT) scan of the abdomen and pelvis was done, which revealed a high-grade mid-ileal small bowel obstruction secondary to right obturator hernia (Figure 2).

**Figure 3:**
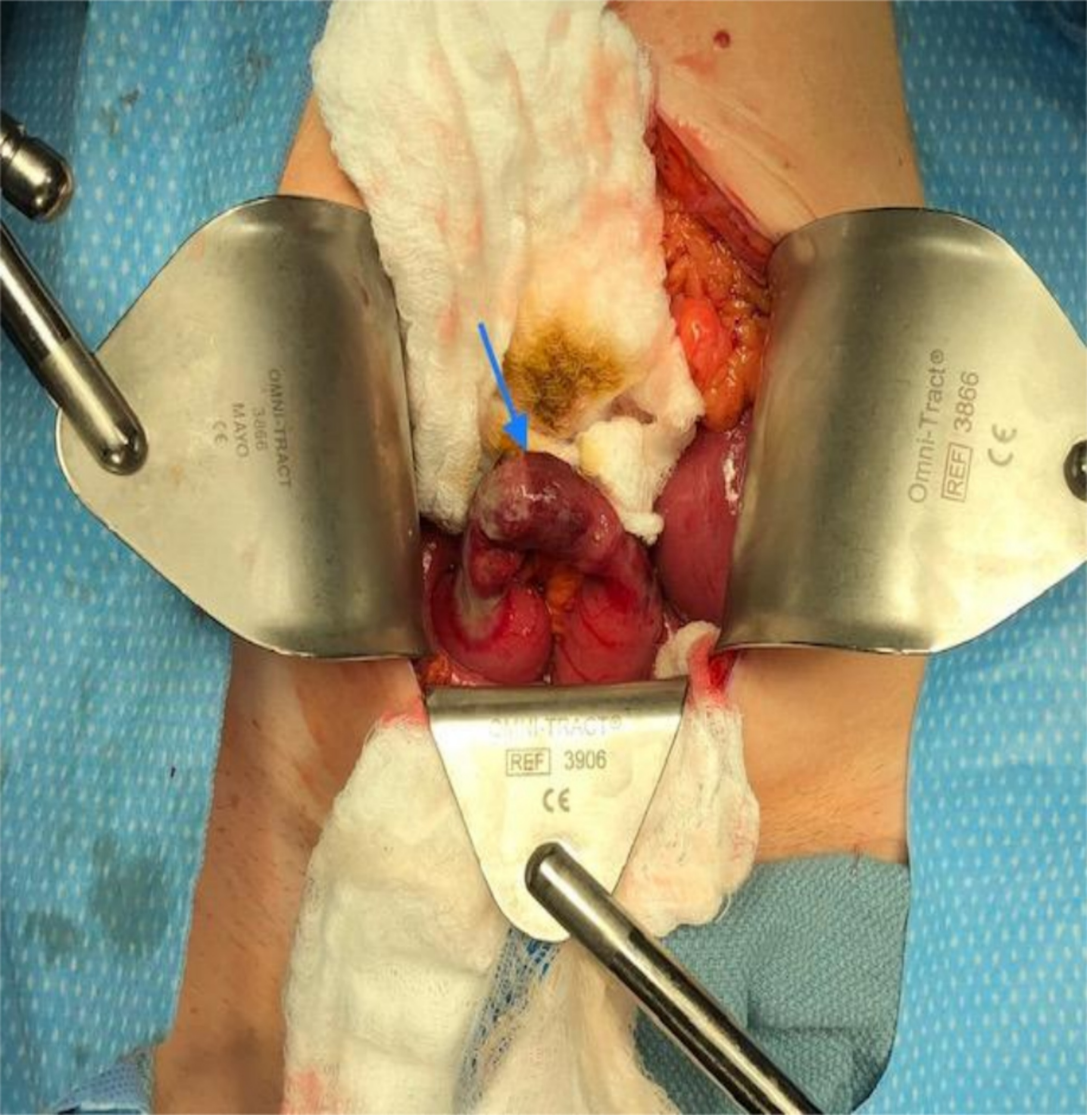
Operative findings Gangrenous loop of the bowel (blue arrow)

Emergent abdomen exploration along with reduction and resection of the herniated ischemic small bowel loop with perforation was performed (Figure 3).

**Figure 4:**
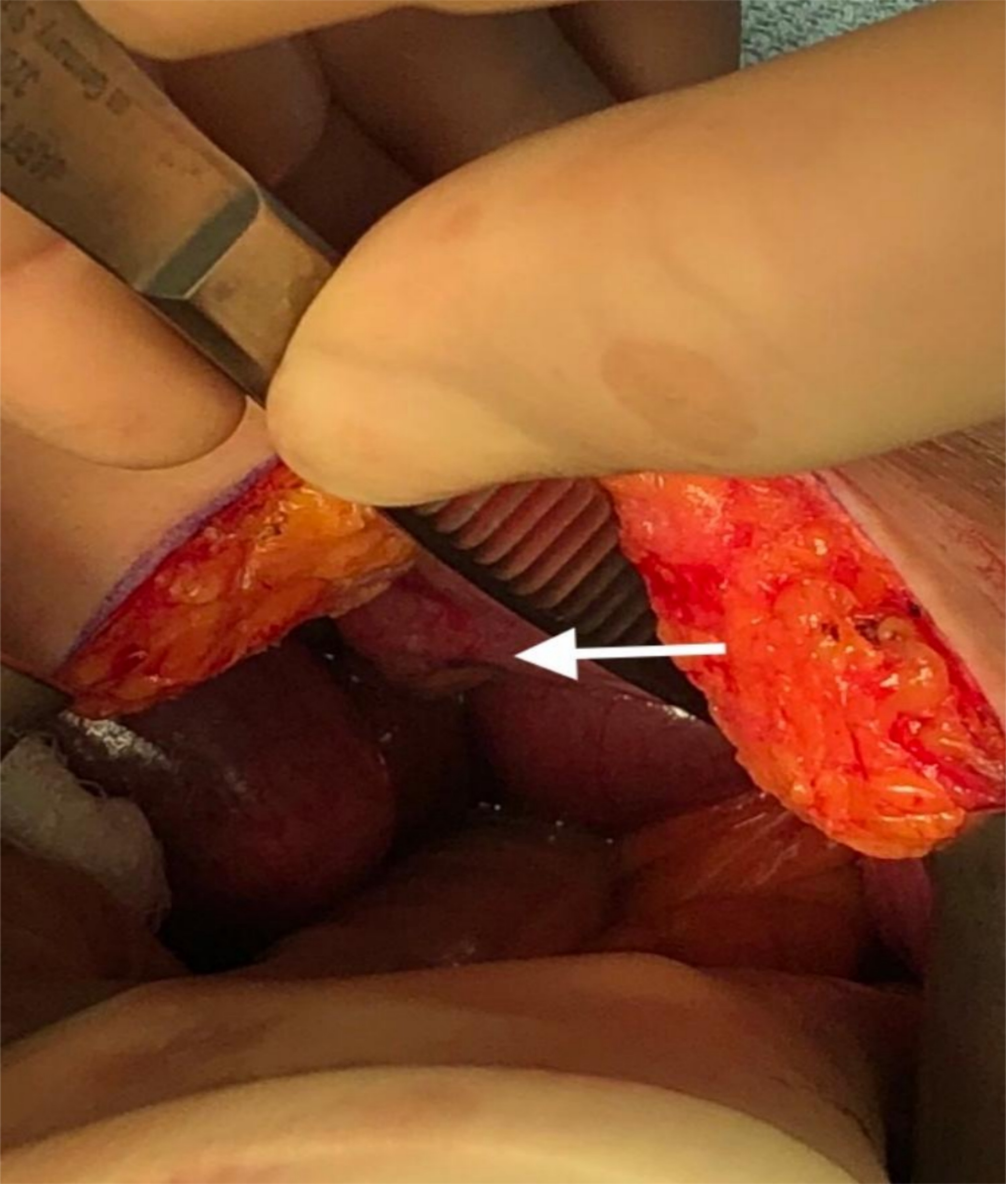
Intraoperative findings Obturator hernia defect prior to repair (white arrow)

The hernia defect (Figure 4) was closed by re-approximating the obturator and pectineal muscles with nonabsorbable nylon sutures. Mesh use was avoided in a contaminated field.

The patient recovered and was discharged from the hospital four days later.

## Discussion

The Howship-Romberg sign was initially described by John Howship back in 1840 [3], and Moritz Heinrich Romberg explained the pathophysiology of an incarcerated obturator hernia. It is present in up to 50% of patients with obturator hernia and is more prevalent when the hernia sac follows the anterior branch of the obturator nerve [4]. The hernia protrudes through the obturator foramen situated bilaterally in the anterolateral pelvic wall, inferior to the acetabulum [5]. Obturator hernias are difficult to diagnose and 80% of patients present with small bowel obstruction symptoms in the form of crampy abdominal pain that can be vague some times, with nausea and vomiting [6]. Symptoms confusing with sciatica has been reported [7]. While flexion of the thigh usually relieves pain in patients with obturator hernia, it has not been reported as a presenting symptom as we describe in our case. Imaging of the bowel herniating through the obturator foramen and lying between the pectineus and obturator muscles is the best diagnostic clue [8-9]. Surgical repair with non-absorbable sutures is the mainstay of therapy performed with open or laparoscopic techniques. A mesh plug has been used in the uncontaminated field. Repair of a contralateral obturator canal defect is rarely attempted due to a low recurrence rate and the need for additional operative time [10].

## Conclusions

Elderly female patients presenting with a positive Howship-Romberg sign and symptoms suggestive of small bowel obstruction should always raise concerns for an obturator hernia. A CT scan of the abdomen and pelvis can be very helpful, and early surgical management is the mainstay of therapy.
